# Two novel *Raoultella* species associated with bleeding cankers of broadleaf hosts, *Raoultella scottia* sp. nov. and *Raoultella lignicola* sp. nov.

**DOI:** 10.3389/fmicb.2024.1386923

**Published:** 2024-05-02

**Authors:** Carrie Brady, Bridget Crampton, Sundeep Kaur, Daniel Maddock, Helene Kile, Dawn Arnold, Sandra Denman

**Affiliations:** ^1^Centre for Research in Bioscience, College of Health, Science and Society, University of the West of England, Bristol, United Kingdom; ^2^Centre for Forest Protection, Forest Research, Farnham, United Kingdom; ^3^Harper Adams University, Newport, United Kingdom

**Keywords:** *Raoultella*, *Enterobacteriaceae*, bleeding canker, *Liriodendron*, *Platanus*, Tilia

## Abstract

Seventeen Gram-negative, facultatively anaerobic bacterial strains were isolated from bleeding cankers of various broadleaf hosts and oak rhizosphere soil in Great Britain. The strains were tentatively identified as belonging to the genus *Raoultella* based on 16S rRNA gene sequencing. Multilocus sequence analysis (MLSA), based on four protein-encoding genes (*fusA*, *leuS*, *pyrG*, and *rpoB*), separated the strains into three clusters within the *Raoultella* genus clade. The majority of strains clustered with the type strain of *Raoultella terrigena*, with the remaining strains divided into two clusters with no known type strain. Whole genome sequencing comparisons confirmed these two clusters of strains as belonging to two novel *Raoultella* species which can be differentiated phenotypically from their current closest phylogenetic relatives. Therefore, two novel species are proposed: *Raoultella scottia* sp. nov. (type strain = BAC 10a-01-01^T^ = LMG 33072^T^ = CCUG 77096^T^) and *Raoultella lignicola* sp. nov. (type strain = TW_WC1a.1^T^ = LMG 33073^T^ = CCUG 77094^T^).

## 1 Introduction

*Raoultella* was proposed as a novel genus in 2001 to contain mostly environmental “*Klebsiella*-like organisms” previously classified as *Klebsiella terrigena*, *Klebsiella ornithinolytica* and *Klebsiella planticola* (Drancourt et al., 2001). In 2014 a fourth species, *Raoultella electrica* isolated from biofilms of a microbial fuel cell, was proposed (Kimura et al., 2014) to bring the total of validly named species to four. There is an ongoing debate and some controversy around the phylogeny of *Raoultella* in relation to *Klebsiella*, with some taxonomists proposing that *Raoultella* should be reunified with *Klebsiella* due to the position of the genus clade within *Klebsiella* in a phylogenomic tree based on core protein sequences (Ma et al., 2021). The GTDB database also lists the *Raoultella* species under the genus *Klebsiella* (Parks et al., 2022), whereas another study suggests that *Klebsiella* is polyphyletic and could be divided into two clades, with *Raoultella* retained as a separate genus (Liu et al., 2020). *Raoultella* are ubiquitous in the environment and isolated from plants, water and soil although there are increasing reports of these species as clinical pathogens, especially *R. planticola* and *R. ornithinolytica* (Sękowska, 2017; Appel et al., 2021). Additionally, *Raoultella* species have been shown to successfully degrade fatty compounds, crude oil and biopolymers, and could play a potential role in protecting the natural environment through the degradation of toxic substances (Sękowska, 2019). For example, a novel *Raoultella* strain (isolated from heavy metal-polluted soil) demonstrated plant growth-promoting characteristics in experiments with pak choi, in addition to its cadmium-binding properties through ion exchange and chelation (Xu et al., 2019). Likewise, an encapsulated *R. planticola* strain applied to cotton seedlings under salt stress was shown to promote germination, biomass and photosynthetic pigment production (Wu et al., 2014). While these examples indicate the positive effects of *Raoultella* species on plant growth, they are predominantly associated with epiphytic lifestyles. Positive effects on plant protection by endophytic *Raoultella* species have been identified, such as the nematicidal activity of *R. ornithinolytica* against the pine wilt nematode (Shanmugam et al., 2018), although these are less frequently reported in literature.

In the present study, 17 strains tentatively identified as *Raoultella* species based on partial 16S rRNA gene sequencing, were isolated from bleeding cankers of various broadleaf hosts and oak rhizosphere soil. The bleeds were sampled as part of a survey of bleeding cankers and wetwood of broadleaf hosts in Great Britain.^1^ Further molecular characterisation of the strains demonstrated that eight of these strains could not be assigned to an existing *Raoultella* species and following a polyphasic approach, were identified as belonging to two novel species for which the names *Raoultella scottia* sp. nov. and *Raoultella lignicola* sp. nov. are proposed.

## 2 Materials and methods

### 2.1 Isolation of bacteria

Bacterial were isolated from exudate, necrotic wood tissue or the rhizosphere soil. Swab samples were collected from exudate of the cankers, or from small bark panels (10 cm × 10 cm) of the bleeding lime trees. Swabs were rehydrated in sterile 1/4 Ringers, spread on Luria-Bertani (LB) agar (Oxoid) and eosin methylene blue (EMB) agar (Merck) and were incubated at 35°C under anaerobic conditions for 4 days. Wood chips were removed from the advancing lesion front of the lime panels with a sterile scalpel, plated on LB agar and incubated at 25°C for 7 days. Single colonies were obtained through re-streaking on LB agar and incubation at 25°C. Bacterial strains were isolated from the rhizosphere soil as previously described (Maddock et al., 2022). A list of strains used in the study along with their isolation source and location is presented in Supplementary Table 1.

### 2.2 Genotypic characterisation

DNA was extracted from all strains with an alkali lysis method (Niemann et al., 1997) followed by storage at −20°C. In order to assign a tentative identification, partial 16S rRNA gene sequencing was performed on all strains using the primer *pD (16F536), and the almost complete 16S rRNA gene was sequenced for the proposed type strains of the novel species using standard primers and PCR conditions (Coenye et al., 1999). Partial 16S rRNA gene sequences were compared against the EzBioCloud database to obtain a tentative identity (Yoon et al., 2017). Multilocus sequence analysis (MLSA) based on partial sequencing of four protein-encoding genes (*fusA*, *leuS*, *pyrG* and *rpoB*) was performed on the strains tentatively identified as *Raoultella* species using the published primers and conditions (Delétoile et al., 2009). Consensus sequences for both the 16S rRNA and protein-encoding genes were generated, aligned and trimmed in BioEdit v7.2.5 (Hall, 1999). The resulting lengths were: 16S rRNA—1,389 bp, *fusA*—633 bp, *leuS*—642 bp, *pyrG*—306 bp and *rpoB*—501 bp. Phylogenetic trees were constructed with the maximum likelihood method and 1,000 bootstrap replicates in PhyML 3.0 (Guindon et al., 2010) following Smart Model Selection (Lefort et al., 2017).

### 2.3 Genomic characterisation

Whole genome sequencing was performed on five representative strains (selected based on clusters observed from the MLSA results) from different hosts and locations: BAC 10a-01-01^T^, Txe 2.2, WB_B2P2.3, TW_WC1a.1^T^ and BAC 2a-02-02. Sequencing was carried out by MicrobesNG (Birmingham, UK) using the Illumina HiSeq platform following DNA extraction by enzymatic cell lysis and purification with SPRI (Solid Phase Reversible Immobilization) beads. Adapters were trimmed from reads using Trimmomatic 0.30 with a sliding window quality cut-off of Q15 (Bolger et al., 2014) and *de novo* assembly was performed using SPAdes version 3.11.1 (Bankevich et al., 2012). The resulting contigs were annotated in the prokaryotic genome annotation pipeline (PGAP) (Tatusova et al., 2016) for GenBank submission, as well as in Prokka (Seemann, 2014).

Pairwise phylogenomic comparisons were carried out between the proposed novel species and existing *Raoultella* species, using genome blast distance phylogeny (GBDP) on the Type (Strain) Genome Server (TYGS) (Meier-Kolthoff and Göker, 2019). Accurate intergenomic distances were inferred under the algorithm “trimming” and distance formula *d*_5_ (Meier-Kolthoff et al., 2013), with 100 distance replicates each. The resulting intergenomic distances were used to infer a balanced minimum evolution tree with branch support via FASTME 2.1.6.1 including SPR postprocessing (Lefort et al., 2015) and 100 pseudo-bootstrap replicates. The tree was rooted at the midpoint (Farris, 1972). DNA-DNA similarity comparisons were conducted with both average nucleotide identity (ANI), using FastANI (Jain et al., 2018), and *in silico* DNA-DNA hybridization (*is*DDH) with the Genome-to-Genome Distance Calculator (GGDC) (Meier-Kolthoff et al., 2022). Average amino acid identity (AAI) and pairwise percentage of conserved proteins (POCP) were calculated using the AAI-matrix calculator from the Kostas Lab (Rodriguez-R and Konstantinidis, 2016) and the script pocp.rb (Hoelzer, 2020) which follows the approach by Qin et al. (2014), respectively.

### 2.4 Genome features

Amino acid sequences from the whole genomes of BAC 10a-01-01^T^, Txe 2.2, WB_B2P2.3 and TW_WC1a.1^T^ were examined to assess the pathogenic potential of their proteome. DIAMOND version v2.1.6 (Buchfink et al., 2021) was used to query the genomes against the Virulence Factor Database (VFDB) (Liu B. et al., 2022), accessed 13th October 2023. A query cut-off of 97% and a percentage identity equal to or greater than 50 were used to ensure high sequence alignment identification (Doonan et al., 2019). To assess the microbe-plant interaction potential of the two novel species, the amino acid annotations were queried against the “plant bacterial only interaction factors” (PIFAR-Pred) and “plant growth-promoting traits” (PGPT-Pred) databases using BlastP + HMMER Aligner/Mapper through the PLant-associated BActeria web resource (PLaBAse) (Martínez-García et al., 2016; Patz et al., 2021; Samad et al., 2022). Following open reading frame identification with orfipy (Singh and Wurtele, 2021), detection of potential type III secretion system effectors (T3SS) was performed using the Effectidor web server (Wagner et al., 2022).

### 2.5 Phenotypic and chemotaxonomic characterisation

Phenotypic and chemotaxonomic characterisation was carried out on all existing *Raoultella* species type strains and BAC 10a-01-01^T^, Txe 2.2, WB_B2P2.3 and TW_WC1a.1^T^ from the present study. Bacterial strains were grown on LB agar for 24 h at 30°C to examine colony morphology, while the temperature range was determined following growth on LB agar at 4, 10, 25, 30, 37 and 41°C for 3 d. Salt and pH tolerance were tested in saline-free nutrient broth supplemented with NaCl (1–11%) in increments of 1% w/v, and in LB broth with the pH adjusted from 5 to 9 in increments of 1 as previously described (Brady et al., 2022). Catalase and oxidase activity were determined by bubble production in 3% v/v H_2_O_2_ and by staining with Kovács reagent (1% tetra-methyl-*p*-phenylenediamine dihydrochloride), respectively. Cell morphology, size and motility were determined using a light microscope and the CellSens software v 1.11 (Olympus Life Science, Tokyo, Japan) following growth in LB broth at 25°C for ∼3 h. The flagella arrangement was visualized by transmission electron microscopy (FEI Tecnai 12 120kV BioTwin Spirit TEM) following negative staining as previously published (Brady et al., 2022).

API 20E, API 50 CHB/E (bioMérieux) and GEN III GN/GP microplate (Biolog) assays were performed on the selected strains as per the manufacturer’s instructions. API 20E and API 50 CHB/E galleries were incubated at 37°C for 24 h and 30°C for 48 h, respectively, while GEN III microplates were incubated at 30°C for 18 h. Fatty acid methyl ester (FAME) profiles were determined by Fera Science Ltd. (York, UK) using the protocol based on the Sherlock Microbial Identification System Version 6.4 (MIDI Inc.). Strains were cultivated on TSA at 28°C for 24 h prior to fatty acid extraction and the results obtained were compared against the RTSBA6 6.21 library.

## 3 Results and discussion

### 3.1 Bacterial isolation

**FIGURE 1:**
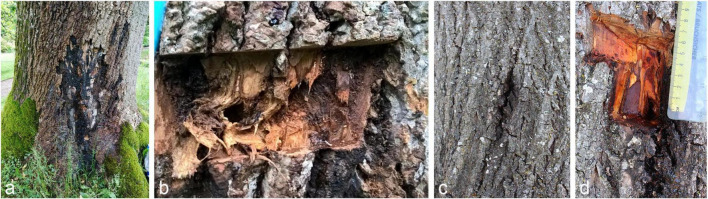
Bleeding cankers of broadleaf hosts. **(a)** External canker symptoms of *Liriodendron tulipifera* at Rosemoor Gardens, Devon. **(b)** Exposed necrotic inner bark of *L. tulipifera*. **(c)** External canker symptoms of *Tilia* x *europaea* at Tidworth Garrison, Wiltshire. **(d)** Exposed necrotic inner bark of *Tilia* x *europaea*.

Strains were isolated over two years (2020–2021) from bleeding cankers of *Liriodendron tulipifera* (tulip tree), *Tilia* x *europaea* (common lime), *Tilia* x *moltkei* (Von Moltke’s lime), *Fagus sylvatica* (common beech) and *Platanus* x *acerifolia* (London plane) (Figure 1) across several counties of Great Britain. External symptoms included dark brown to black bleeding exudate, staining the outer bark while the underlying inner bark was necrotic and stained rust to brown in color. Additionally, several strains were isolated from the rhizosphere soil of both healthy and diseased oak suffering from Acute Oak Decline in Surrey, Great Britain.

### 3.2 Genotypic characterisation

**FIGURE 2:**
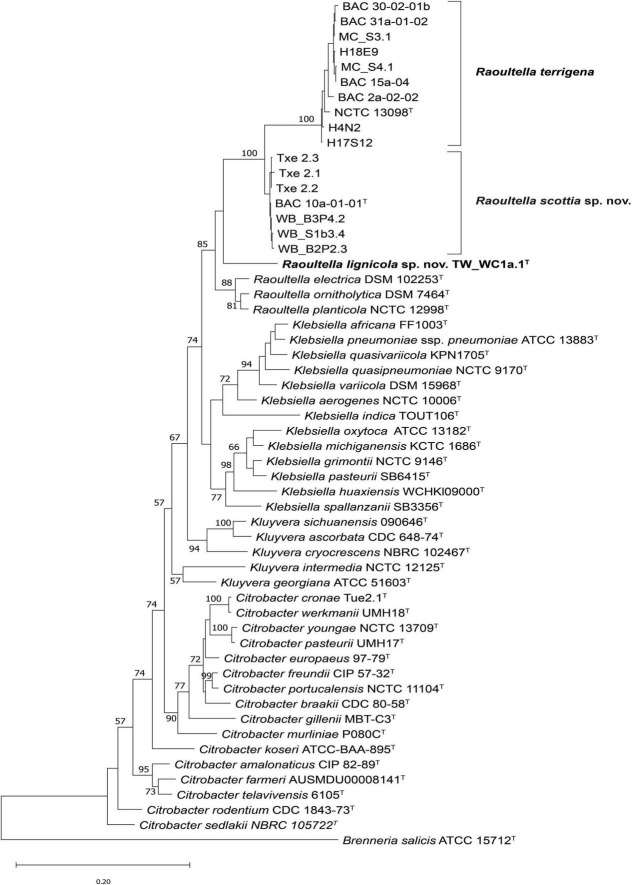
Maximum likelihood tree based on concatenated partial *fusA*, *leuS*, *pyrG* and *rpoB* gene sequences of *Raoultella scottia* sp. nov., *Raoultella lignicola* sp. nov. and current closest phylogenetic relatives. Bootstrap values after 1,000 replicates are expressed as percentages (values > 50% shown). *Brenneria salicis* (ATCC 15712^T^) is included as an outgroup. The scale bar indicates the fraction of substitutions per site. ^T^ = type strain.

Pairwise similarity values for the partial 16S rRNA gene sequences of the strains demonstrated highest similarity to *R. terrigena* (99.3–100%), and to *Klebsiella*, *Kluyvera*, *Citrobacter* and *Lelliottia* species (∼99.0%). In the maximum likelihood tree based on concatenated partial sequences of *fusA*, *leuS*, *pyrG* and *rpoB* genes, the strains tentatively identified as *Raoultella* species formed three clusters (Figure 2). A large proportion of strains, including those isolated from London plane, oak, beech, lime and rhizosphere soil, clustered with the type strain of *Raoultella terrigena* with 100% bootstrap support. The remaining strains were divided into two clusters, which were situated between the *R. terrigena* cluster and the other existing *Raoultella* species. The first contained seven strains from tulip and lime trees and clustered with 100% bootstrap support, basal to the *R. terrigena* cluster. The second contained a single strain isolated from lime (TW_WC1a.1^T^) on its own branch, although without significant bootstrap support. Neither of these two clusters contained any reference strains, suggesting they belong to two novel species. The 16S rRNA gene tree (Supplementary Figure 1) demonstrated a polyphyletic topology for the genus *Raoultella*, with *R. terrigena* clustering between strains of one of the proposed novel species, and the remaining *Raoultella* species clustering at a lower level with the proposed single strain species, although there is little to no bootstrap support for these clusters. It should be noted that the 16S rRNA gene Sanger sequence for BAC 10a-01-01^T^ differa from that produced from the whole genome sequence by several base pairs. However, *Raoultella* species are known to have approximately eight copies of the 16S rRNA gene (Stoddard et al., 2015) and it has been previously demonstrated that sequence differences can exist between copies, especially within the Proteobacteria (Ibal et al., 2019) which may account for these nucleotide differences.

### 3.3 Genomic characterisation

**FIGURE 3:**
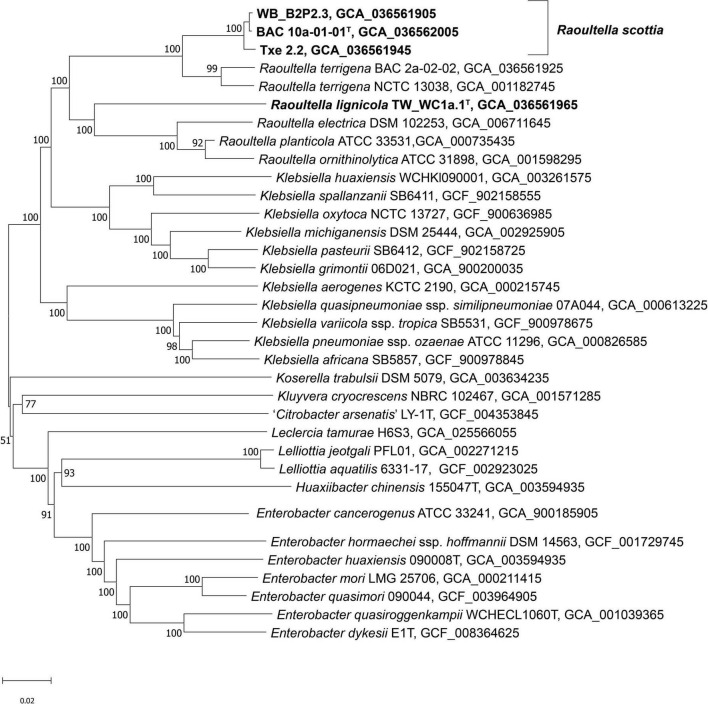
Phylogenomic tree of *Raoultella scottia* sp. nov., *Raoultella lignicola* sp. nov. and phylogenetic relatives. GBDP pseudo-bootstrap support values > 60% shown at the nodes (from 100 replicates), with an average branch support of 85.4%. The branch lengths are scaled in terms of GBDP distance formula *d*_5_ and the tree is rooted at the midpoint. ^T^ = type strain.

Genome sequences were submitted to GenBank under the BioProject number PRJNA955701. Assembly accession numbers and genome metrics are listed in Supplementary Table 2. Prokka annotation of the sequences resulted in genomes of 4.39–5.93 Mbp, with a G + C content of 56.0 to 57.5 mol %. The phylogenomic tree presents a clear phylogeny for the genus *Raoultella* with current species and proposed novel species all contained in a well-supported clade (Figure 3). BAC 10a-01-01^T^, WB_B2P2.3 and Txe 2.2 formed a single distinct cluster, with *R. terrigena* indicated as the current closest phylogenetic neighbor, while TW_WC1a.1^T^ clustered with the remaining *Raoultella* species at a lower level but also with 100% support. Results from the phylogenomic analyses agree with those of Liu et al. (2020), with species of the genus *Klebsiella* divided into two clades and *Raoultella* species constituting their own discrete clade. Furthermore, the topology of the phylogenomic tree is congruent with a previous phylogeny based on non-paralogous ribosomal MLST loci (Bray et al., 2022) where the genus *Raoultella* is shown to separate *Klebsiella* into two clades, the “*pneumoniae-africana-variicola*” clade and the “*oxytoca-indica-grimontii-michiganensis*” clade.

The ANI and *is*DDH values (Table 1) confirmed that BAC 10a-01-01^T^, WB_B2P2.3 and Txe 2.2 belong to a single taxon with values exceeding 98.9 and 92.8%, respectively, which are well above the suggested species boundary cut-off values (Goris et al., 2007; Meier-Kolthoff et al., 2013). Additionally, both the ANI and *is*DDH values were higher between *R. terrigena* and the first proposed novel species reflecting their close phylogenetic relationship. TW_WC1a.1^T^, comprising the second proposed novel species, displayed ANI values of 85.6–87.6% to the first proposed novel species and the existing *Raoultella* species.

**TABLE 1:** DNA-DNA similarity values between *Raoultella scottia* nov. sp. nov., *Raoultella lignicola* sp. nov. and existing species of the genus *Raoultella* based on average nucleotide identity (fastANI—lower left) and *in silico* DNA-DNA hybridisation (*is*DDH—upper right).

*is*DDH FastANI	1	2	3	4	5	6	7	8	9
1	100	94.1	97.9	56.9	56.4	29.1	30.1	30.4	30.2
2	99.1	100	92.8	56.5	56.4	29.0	30.0	30.4	30.0
3	99.7	98.9	100	57.2	56.5	29.4	30.2	30.7	30.2
4	94.1	94.1	94.1	100	76.7	29.1	29.9	30.3	29.9
5	94.1	94.1	94.1	97.0	100	29.1	29.6	30.0	29.6
6	85.6	85.6	85.7	85.9	85.7	100	32.6	32.8	32.6
7	85.9	85.9	85.9	86.0	85.9	87.4	100	53.6	66.3
8	86.0	86.1	86.2	86.1	86.0	87.4	93.5	100	55.4
9	86.1	86.1	86.2	86.1	86.1	87.6	95.7	93.9	100

1 = *Raoultella scottia* BAC 10a-01-01^T^ (GCA_036562005), 2 = *Raoultella scottia* Txe 2.2 (GCA_036561945), 3 = *Raoultella scottia* = WB_B2P2.3 (GCA_036561905), 4 = *Raoultella terrigena* NBRC 14941^T^ (GCA_006539725), 5 = *Raoultella terrigena* BAC 2a-02-02 (GCA_036561925), 6 = *Raoultella lignicola* TW_WC1a.1^T^ (GCA_036561965), 7 = *Raoultella planticola* ATCC 33521^T^ (GCA_000735435), 8 = *Raoultella electrica* DSM 102253^T^ (GCA_006711645), 9 = *Raoultella ornithinolytica* NBRC 105727^T^ (GCA_013457875), ^T^ = type strain. Percentages above cut-off value for species delimitation (> 95% for ANI and > 70% for *is*DDH) are shaded.

Intra-genus AAI values of > 90% were observed between the proposed novel species and existing *Raoultella* species (Supplementary Table 3). Four representative *Klebsiella* species were included in the AAI and POCP analyses. The AAI values between *Raoultella* species, including the proposed novel species, and *Klebsiella* species were in the range of 87.4–89.9%. Several previous studies have used AAI to delineate genera with suggested boundary cut-off values, however, these do seem to differ between bacterial families (Walter et al., 2017; Wirth and Whitman, 2018; Nicholson et al., 2020; Zheng et al., 2020) and there is no single boundary that can be applied across all families and genera. Based on the values observed in the present study, the boundary cut-off value for the *Enterobacteriaceae* could be substantially higher than the 74–79% proposed for other families (Nicholson et al., 2020; Soutar and Stavrinides, 2022). The POCP values were less informative in terms of genus delineation as they ranged from 65 to 89.9% within *Raoultella*, 40.5 to 79.8% between *Raoultella* and *Klebsiella* species, and 46.7 to 84.0% within *Klebsiella*.

### 3.4 Genome features

Queries of the amino acid sequences against the VFDB revealed that several virulence genes are present in both novel species. The majority of these are associated with adherence, effector delivery systems, immune modulation and nutritional/metabolic factors. Genes for the type II, IV and VI secretion systems were found in the first novel *Raoultella* species (BAC 10a-01-01^T^, Txe 2.2 and WB_B2P2.3) while only genes for the type II secretion system were found in the second novel *Raoultella* species (TW_WC1a.1^T^). Results from the genome features queries are listed in Supplementary Table 4. The lack of type III secretion system genes for both species was confirmed by the results from Effectidor. The absence of a type III secretion system, and the relatively low numbers of virulence genes for biofilm formation, invasion and response regulation coupled with the low percentage of pectate cell wall degrading enzymes and lipopolysaccharides suggest that the two novel species do not have high pathogenic potential. Furthermore, the identified gene categories have a limited association to plant hosts. For example, many of the genes associated with immune modulation such as *lpx* genes that significantly contribute to lipopolysaccharide biosynthesis (Moosavian et al., 2020) are homologous to those experimentally identified in human pathogens such as *Haemophilus influenzae*. This could potentially be seen as a limitation of the VFDB, however, based on the presence of important plant pathogens such as *P. syringae* in the dataset it would seem more likely that the novel species analyzed in this work have limited ability to interact with their host. Likewise, the Hcp, clpV and dotU type VI secretion proteins identified here show the highest homology to those experimentally verified in *Klebsiella pneumoniae*. However, this is likely due to evolutionary lineage and does not limit the importance of these type VI secretion system genes which are known to play crucial roles in colonization, growth, competition, virulence, biofilm formation and environmental iron acquisition in plant pathogens such as *Acidovorax citrulli* and *Xanthomonas phaseoli* pv. *manihotis* (Montenegro Benavides et al., 2021; Fei et al., 2022). While the novel species identified in this work may not resemble plant pathogens, they still appear to have homologous genes required for colonization of the plant and/or lesion.

Relatively low percentages of plant growth-promoting traits also suggest that the two novel species do not play a significant role in improving plant nutrition and, as they were isolated from the lesion microbiome, this scenario is unlikely.

While the identified virulence genes limit the pathogenic potential of the two novel species, they do, however, highlight an interesting comparison. *R. terrigena* has previously been isolated from both the gut of ghost moth larvae (*Thitarodes xiaojinensis*), and from root and seed tissue of alpine bistort (*Polygonum viviparum*), which acts as the host plant during larvae maturation. *R. terrigena* was shown to degrade quercetin produced by the plant, enabling the growth of another moth larvae species which is normally inhibited by this secondary metabolite (Liu G. et al., 2022). With many of the virulence features identified in the two novel *Raoultella* species being related to immune modulation and nutritional/metabolic factors, the potential for a similar role for these bacteria and herbivorous insects that feed on weakened host plants should not be ignored. This model could be of importance when considering the correlation of *Agrilus biguttatus* and oak, a plant-pathogen-pest interaction in which a healthy host is able to limit larval development but a weakened host suffering from Acute Oak Decline is not (Brown et al., 2017).

### 3.5 Phenotypic and chemotaxonomic characterisation

Following incubation on LB agar at 30°C for 24 h, colonies were round, smooth with entire edges, 3–4 mm in diameter, slightly convex, translucent, non-pigmented and cream in color. Strains exhibited weak growth at 10°C but grew readily at 25–37°C, and only *R. planticola* and *R. ornithinolytica* grew well at 41°C. Strains from all *Raoultella* species grew in the pH range of 5–9 and could tolerate salt in supplemented broth up to 8% and weakly at 9–10%. All species were positive for catalase and negative for oxidase activity. Cells from both novel species are short rods (1.0–1.3 μm × 1.6–2.6 μm) and are found singularly, in pairs or rarely in chains, and are non-motile but possess fimbriae (Supplementary Figure 2).

The two proposed novel species could be differentiated from each other, and existing members of *Raoultella*, by their reactions to acetoin production, ornithine decarboxylase and urease, as well as the utilization of several carbon sources. Distinguishing characteristics for the genus are listed in Table 2, and a list of carbon sources utilized by all current members of *Raoultella* is presented in Supplementary Table 5. The fatty acid composition for the two proposed novel species were similar to those of the existing *Raoultella* species, with C_16:0_, C_18:1_ ω7*c* and summed feature 3 (C_16:1_ ω7*c* and/or C_16:1_ ω6*c*) comprising the major fatty acids. Complete fatty acid profiles are presented in Table 3.

**TABLE 2:** Phenotypic characteristics that can distinguish *Raoultella scottia* sp. nov. and *Raoultella lignicola* sp. nov. from existing members of the genus *Raoultella.*

Characteristic	*Raoultella scottia* *n* = 3	*Raoultella lignicola* TW-WC1.a.1^T^	*Raoultella terrigena* LMG 3222^T^	*Raoultella planticola* CCUG 15718^T^	*Raoultella electrica* CCUG 70816^T^	*Raoultella ornithinolytica* CCUG 26769^T^
Acetoin production	+	−	+	+	+	+
Ornithine decarboxylase	+	−	−	−	−	+
Urease	−	−	−	+	+	+
**Acid production from (API 50 CBH/E):**						
Dulcitol	v*^a^*	+	−	−	−	−
Amygdalin	−	−	−	+	+	+
D-melezitose	−	−	+	−	+	−
Starch	−	−	−	−	+	+
D-tagatose	v*^a^*	+	−	−	−	−
Potassium gluconate	+	+	−	−	−	+
**Utilization of (Biolog):**						
D-maltose	v*^a^*	−	−	−	+	+
Stachyose	v*^a^*	+	−	+	+	+
D-arabitol	v*^a^*	+	−	+	+	+
D-lactic acid	−	+	−	+	+	−
**Sensitivity to (Biolog):**						
Fusidic acid	v*^a^*	+	+	+	+	−
Potassium tellurite	v*^a^*	+	+	+	+	+

+, 90–100% strains +; −, 91–100% strains −; v, variable *^a^*Type strain positive.

**TABLE 3:** Major fatty acid composition (percentage of peak areas) of *Raoultella scottia* sp. nov., *Raoultella lignicola* sp. nov. and existing members of the genus *Raoultella*.

Fatty acid	*Raoultella scottia* *n* = 3	*Raoultella lignicola* TW-WC1.a.1^T^	*Raoultella terrigena* LMG 3222^T^	*Raoultella planticola* CCUG 15718^T^	*Raoultella electrica* CCUG 70816^T^	*Raoultella ornithinolytica* CCUG 26769^T^
**Saturated fatty acids**						
C_12:0_	3.5 (± 0.1)	3.6	2.4	3.6	3.8	4.0
C_14:0_	6.6 (± 0.7)	7.2	5.6	6.1	8.3	7.7
C_16:0_	29.7 (± 0.8)	25.8	28.0	28.5	30.7	28.8
**Unsaturated fatty acids**						
C_18:1_ ω7*c*	20.7 (± 0.8)	23.9	21.1	24.9	19.7	21.8
**Cyclopropane fatty acids**						
C_17:0_	8.8 (± 0.8)	5.8	14.0	7.2	9.5	7.3
**Summed features**						
2: C_14:0_ 3-OH and/or iso-C_16:1_	8.4 (± 0.3)	8.6	8.4	7.8	7.7	7.3
3: C_16:1_ ω7*c* and/or C_16:1_ ω6*c*	20.8 (± 1.8)	23.0	19.1	19.5	18.1	21.3

## 4 Conclusion

Despite the role of several *Raoultella* species in causing human infections including urinary tract infections, sepsis and bacteraemia (Kaya et al., 2015; Seng et al., 2016; Mihu et al., 2023) there is a clear association of this genus with the natural environment with *R. planticola* and *R. terrigena* strains often isolated from botanical, aquatic and soil environments (Grimont and Grimont, 2015). The isolation of two novel *Raoultella* species associated with bleeding cankers of broadleaf hosts reinforces this association. Due to the low number of identified virulence factors in both novel species, and their sporadic isolation from bleeding cankers in various tree species, it is unlikely that they play a role in the symptom development. Instead, it is likely that they are part of the tree hosts’ microbiome or wider natural environment.

The future of the genus *Raoultella* is currently unclear as taxonomists fail to agree on whether the genus *Klebsiella* is polyphyletic, or monophyletic with the inclusion of *Raoultella* in the *Klebsiella* genus clade. There is little to differentiate these two genera based on phenotype or ecology, and their phylogeny varies depending on whether it is based on core genome sequences, core protein sequences or rMLST loci (Liu et al., 2020; Ma et al., 2021; Bray et al., 2022). As additional novel species are classified as belonging to either *Raoultella* or *Klebsiella*, the true taxonomy of these genera may become more apparent. The identification of two novel *Raoultella* species in the present study based on phylogenetic, genomic and phenotypic data, provides support for the genus to remain independent from *Klebsiella* for the time being. The names *Raoultella scottia* sp. nov. (type strain = BAC 10a-01-01^T^ = LMG 33072^T^ = CCUG 77096^T^) and *Raoultella lignicola* sp. nov. (type strain = TW_WC1a.1^T^ = LMG 33073^T^ = CCUG 77094^T^) are proposed for the strains isolated from bleeding cankers of broadleaf hosts. The description of *Raoultella lignicola* sp. nov. is currently based on a single strain and, as additional strains are isolated, the biochemical profile may change.

### 4.1 Emended description of the genus *Raoultella* (Drancourt et al., 2001)

*Raoultella* (Ra.oul.tel’la. M.L. dim. suff. *tella*; N.L. fem. dim. n. *Raoultella*, named after the French bacteriologist Didier Raoult, Université de la Méditerranée, Marseille, France)

The description is based on data from Drancourt et al. (2001), Kimura et al. (2014) and this study.

Gram-negative short capsulated rods with rounded ends (0.9–1.3 μm × 1.6–2.6 μm), facultatively anaerobic, oxidase-negative and catalase positive. Cells occur singularly, in pairs and sometimes in chains, non-motile but possess fimbriae. On LB agar, colonies are cream-colored, round, smooth with entire edges, 3–4 mm in diameter, slightly convex, and translucent but form large mucoid colonies on McConkey agar. Strains can grow at temperatures between 10 and 37°C, growth at 41°C is variable depending on the species. Strains can grow within the pH range 5–9 and in the presence of 0–8% (w/v) NaCl. Positive for β-galactosidase activity, lysine decarboxylase and citrate utilization, but negative for H_2_S production and tryptophan deaminase and gelatinase activity. Arginine dihydrolase, ornithine decarboxylase and urease activity are variable along with acetoin and indole production. Nitrate is reduced to nitrite. Utilize glucose, mannose, fructose, galactose, D-glucose-6-phosphate and D-fructose-6-phosphate.

Major fatty acids include C_16:0_, C_18:1_ ω7*c* and summed feature 3 (C_16:1_ ω7*c* and/or C_16:1_ ω6*c*).

Frequently isolated from environmental sources including water, soil, plants and woody tissues of broadleaf trees. Several species are considered opportunistic pathogens and cause pneumonia and bacteraemia in oncologic and immune-compromised patients.


The⁢G+C⁢content⁢ranges⁢from⁢54.1⁢to⁢58.0⁢mol%.

The type species is *Raoultella planticola*.

### 4.2 Description of *Raoultella scottia* sp. nov.

*Raoultella scottia* (scot’ti.a. N.L. fem. n. *scottia*, named after Lewis Scott for his contribution and support of Acute Oak Decline research.)

The description is as given for the genus with the following additional characteristics. Negative for arginine dihydrolase, urease activity and indole production, but positive for ornithine decarboxylase and acetoin production. Acid is produced from glycerol, L-arabinose, D-ribose, D-xylose, D-adonitol, D-galactose, D-glucose, D-fructose, D-mannose, L-sorbose, inositol, D-mannitol, D-sorbitol, methyl-αD-glucopyranoside, N-acetylglucosamine, arbutin, esculin ferric citrate, salicin, D-cellobiose, D-maltose, D-lactose, D-melibiose, D-saccharose, D-trehalose, D-raffinose, gentiobiose, L-fucose, D-arabitol, potassium gluconate and potassium 5-ketogluconate; while production from dulcitol and D-tagatose is variable.


The⁢DNA⁢G+C⁢content⁢ranges⁢from⁢56.6⁢to⁢57.2⁢mol%.

The type strain is BAC 10a-01-01^T^ = LMG 33072^T^ = CCUG 77096^T^, and was isolated from a bleeding canker of *Liriodendron tulipifera* (tulip tree) in Great Britain.

Accession numbers for the whole genome and 16S rRNA gene sequences are JARXNH000000000 and OQ813756, respectively.

### 4.3 Description of *Raoultella lignicola* sp. nov.

*Raoultella lignicola* (li.gni.co.la. L. masc./fem. n. suff. *-cola*, dweller; N.L. masc./fem. n. *lignicola*, wood inhabitant.)

The description is as given for the genus with the following additional characteristics. Negative for arginine dihydrolase, ornithine decarboxylase and urease activity, and indole and acetoin production. Acid is produced from glycerol, L-arabinose, D-ribose, D-xylose, D-adonitol, D-galactose, D-glucose, D-fructose, D-mannose, L-sorbose, inositol, D-mannitol, D-sorbitol, methyl-αD-glucopyranoside, *N*-acetylglucosamine, arbutin, esculin ferric citrate, salicin, D-cellobiose, D-maltose, D-lactose, D-melibiose, D-saccharose, D-trehalose, D-raffinose, gentiobiose, L-fucose, D-arabitol, potassium gluconate and potassium 5-ketogluconate.


The⁢DNA⁢G+C⁢content⁢is⁢56.0⁢mol%.

The type strain is TW_WC1a.1^T^ = LMG 33073^T^ = CCUG 77094^T^, and was isolated from a bleeding canker of *Tilia* x *europaea* (common lime) in Great Britain.

Accession numbers for the whole genome and 16S rRNA gene sequences are JARXNK000000000 and OQ813758, respectively.

## Data availability statement

The datasets presented in this study can be found in online repositories. The names of the repository/repositories and accession number(s) can be found below: https://www.ncbi.nlm.nih.gov/genbank/, OQ813756 - OQ813758; https://www.ncbi.nlm.nih.gov/genbank/, OQ829282 - OQ829349; https://www.ncbi.nlm.nih.gov/genbank/, JARXNH000000000 - JARXNL000000000.

## Author contributions

CB: Conceptualization, Data curation, Formal analysis, Investigation, Methodology, Project administration, Resources, Validation, Writing – original draft, Writing – review & editing. BC: Data curation, Investigation, Writing – review & editing. SK: Data curation, Investigation, Writing – review & editing. DM: Data curation, Formal analysis, Investigation, Writing – review & editing. HK: Data curation, Investigation, Writing – review & editing. DA: Funding acquisition, Writing – review & editing. SD: Funding acquisition, Writing – review & editing.

## Footnotes

1
https://bacterialplantdiseases.uk/bac-stop/
