# Supplementary material for: Two novel Raoultella species associated with bleeding cankers of broadleaf hosts, Raoultella scottia sp. nov. and Raoultella lignicola sp. nov
Source: Front Microbiol. 2024 May 2;15:1386923. doi: 10.3389/fmicb.2024.1386923 (PMC11096500; doi:10.3389/fmicb.2024.1386923)

Supplementary Material

Supplementary Table 1: Strains of *Raoultella scottia* sp. nov., *Raoultella lignicola* sp. nov. and *Raoultella terrigena* investigated in this study

Strain	Location	Grid reference	Year of isolation	Source	*fusA**	*leuS**	*pyrG**	*rpoB**
Raoultella scottia								
BAC 10a-01-01^T	Rosemoor Gardens, Devon, Great Britain	SS 49900 17600	2020	*Liriodendron tulipifera*, swab from exudate	OQ829291	OQ829308	OQ829325	OQ829342
Txe 2.1	Tidworth Garrison, Wiltshire Great Britain	SU 22683 48180	2020	*Tilia* x *europaea*, inner bark lesion	OQ829292	OQ829309	OQ829326	OQ829343
Txe 2.2	Tidworth Garrison, Wiltshire Great Britain	SU 22683 48180	2020	*Tilia* x *europaea*, inner bark lesion	OQ829293	OQ829310	OQ829327	OQ829344
Txe 2.3	Tidworth Garrison, Wiltshire Great Britain	SU 22683 48180	2020	*Tilia* x *europaea*, inner bark lesion	OQ829294	OQ829311	OQ829328	OQ829345
WB_B2P2.3	Westonbirt Arboretum, Gloucestershire, Great Britain	ST 84606 89691	2021	*Tilia* x *moltkei*, inner bark lesion	OQ829295	OQ829312	OQ829329	OQ829346
WB_B3P4.2	Westonbirt Arboretum, Gloucestershire, Great Britain	ST 84606 89691	2021	*Tilia* x *moltkei*, inner bark lesion	OQ829296	OQ829313	OQ829330	OQ829347
WB_S1b3.4	Westonbirt Arboretum, Gloucestershire, Great Britain	ST 84606 89691	2021	*Tilia* x *moltkei*, swab from exudate	OQ829297	OQ829314	OQ829331	OQ829348
Raoultella lignicola								
TW_WC1a.1^T	Tidworth Garrison, Wiltshire Great Britain	SU 22683 48180	2021	*Tilia* x *europaea*, inner bark lesion	OQ829298	OQ829315	OQ829332	OQ829349
Raoultella terrigena								
BAC 2a-02-02	St James Park, London, Great Britain	TQ 29722 80095	2020	*Platanus* x *acerifolia*, swab from exudate	OQ829282	OQ829299	OQ829316	OQ829333
BAC 15a-04	St James Park, London, Great Britain	TQ 29694 80044	2020	*Platanus* x *acerifolia*, swab from exudate	OQ829283	OQ829300	OQ829317	OQ829334
BAC 30-02-01b	Uckfield, East Sussex, Great Britain	TQ 47133 20330	2021	*Quercus* sp., swab from exudate	OQ829284	OQ829301	OQ829318	OQ829335

BAC 31a-01-02	Bulford, Wiltshire, Great Britain	SU 19682 44211	2021	*Fagus sylvatica*, swab from exudate	OQ829285	OQ829302	OQ829319	OQ829336
H4N2	Hatchlands Park, Surrey, Great Britain	TQ 07108 52296	2020	*Quercus robur* (healthy), rhizosphere soil	OQ829286	OQ829303	OQ829320	OQ829337
H17S12	Hatchlands Park, Surrey, Great Britain	TQ 06915 52423	2020	*Quercus robur* (AOD), rhizosphere soil	OQ829287	OQ829304	OQ829321	OQ829338
H18E9	Hatchlands Park, Surrey, Great Britain	TQ 06931 52452	2020	*Quercus robur* (healthy), rhizosphere soil	OQ829288	OQ829305	OQ829322	OQ829339
MC_S3.1	Minchinhampton, Gloucestershire, Great Britain	SO 87208 00890	2021	*Tilia* x *europaea*, swab from exudate	OQ829289	OQ829306	OQ829323	OQ829340
MC_S4.1	Minchinhampton, Gloucestershire, Great Britain	SO 87208 00890	2021	*Tilia* x *europaea*, swab from exudate	OQ829290	OQ829307	OQ829324	OQ829341

^{T} = type strain

* GenBank accession numbers

Supplementary Table 2: Whole genome sequence information of *Raoultella* strains investigated in this study

Strain	Genbank assembly accession	Biosample	Size (Mbp)	Number of contigs (with PEGs)	N50	Number of coding sequences	Number of RNAs	GC content (mol %)
BAC 10a-01-01^{T}	GCA_036562005	SAMN34185977	4.73	57	314 172	4 671	52	56.6
Txe 2.2	GCA_036561945	SAMN34185978	4.99	28	842 031	4 740	83	57.2
WB_B2P2.3	GCA_036561905	SAMN34185979	4.39	49	218 453	4 324	49	56.7

TW_WC1a.1^{T}	GCA_036561965	SAMN34185980	5.43	148	610 980	5 218	90	56.0
BAC 2a-02-02	GCA_036561925	SAMN34185981	5.93	170	654 524	5 725	107	57.5

Supplementary Table 3: Average amino acid identity (AAI) values (bottom right) and percentage of conserved proteins (POCP) (top left) between *Raoultella scottia* nov. sp. nov., *Raoultella lignicola* sp. nov., existing species of the genus *Raoultella* and selected species of *Klebsiella*

	**POCP**												
**AAI**	**1**	**2**	**3**	**4**	**5**	**6**	**7**	**8**	**9**	**10**	**11**	**12**	**13**
**1**	100	79.4	83.2	76.8	78.9	74.1	74.7	70.6	75.6	42.7	69.8	69.4	69.8
**2**	99.0	100	74.6	79.4	81.2	75.2	76.8	72.3	77.9	43.9	71.9	71.6	73.1
**3**	99.6	98.7	100	71.7	74.4	70.0	70.0	65.0	71.1	40.5	65.2	65.6	65.6
**4**	96.0	96.2	96.0	100	88.1	82.6	84.6	78.4	85.5	48.9	80.7	76.9	79.6
**5**	96.0	96.2	96.0	97.9	100	83.5	84.6	76.2	84.8	ND	ND	ND	ND
**6**	90.3	90.2	90.0	90.5	90.3	100	83.6	74.7	83.4	47.8	78.3	77.2	78.5
**7**	90.4	90.4	90.2	90.5	90.4	92.4	100	80.5	89.9	48.7	79.7	77.3	79.4
**8**	90.2	90.3	90.0	90.7	90.6	92.5	95.8	100	80.8	45.5	74.5	72.3	73.4
**9**	90.5	90.7	90.3	90.9	90.6	92.5	97.5	96.2	100	49.3	78.8	78.8	79.8
**10**	87.8	87.7	87.5	88.0	ND	88.5	88.2	88.3	88.5	100	52.9	46.7	49.6
**11**	87.8	87.7	87.4	87.9	ND	88.2	88.2	88.2	88.5	97.5	100	76.9	81.3
**12**	89.4	89.3	88.9	89.7	ND	89.4	89.6	89.6	89.8	88.4	88.6	100	84.0
**13**	89.3	89.1	89.0	89.3	ND	89.3	89.4	89.5	89.5	88.7	88.7	88.6	100

1 = *Raoultella scottia* BAC 10a-01-01^T (GCA_036562005), 2 = *Raoultella scottia* Txe 2.2 (GCA_036561945), 3 = *Raoultella scottia* WB_B2P2.3 (GCA_036561905), 4 = *Raoultella terrigena* NBRC 14941^T (GCA_006539725), 5 = *Raoultella terrigena* BAC 2a-02-02 (GCA_036561925), 6 = *Raoultella lignicola* TW_WC1a.1^T (GCA_036561965), 7 = *Raoultella planticola* ATCC 33521^T (GCA_000735435), 8 = *Raoultella electrica* DSM 102253^T (GCA_006711645), 9 = *Raoultella ornitholytica* NBRC 105727^T (GCA_013457875), 10 = *Klebsiella pneumoniae* DSM 30104^T (GCA_000281755), 11 = *Klebsiella africana* 200023^T (GCA_020526085), 12 = *Klebsiella huaxiensis* WCHKl090001^T (GCA_003261575), 13 = *Klebsiella oxytoca* DSM 5175^T (GCA_020115535), ND = not determined, ^{T} = type strain

Supplementary Table 4: Proportion of notable virulence genes and plant interaction factors computationally identified from novel species of *Raoultella*. Type strains are listed with number of virulence genes from the comparison of the proteome to the VFDB indicating their pathogenic potential, the percentage of plant interaction factors identified via PIFAR-Pred and the percentage of plant growth-promoting traits identified via PGPT-Pred.

**Number of virulence genes**	***Raoultella scottia* BAC 10a-01-01^{T}**	***Raoultella scottia* Txe 2.2**	***Raoultella scottia* WB_B2P2.3**	***Raoultella lignicola* TW_WC1a.1^{T}**
Adherence	27	25	10	34
Antimicrobial activity/Competitive advantage	6	5	4	7
Biofilm	9	11	1	11
Effector delivery system	18	24	9	10
Exotoxin	1	1	1	0
Invasion	1	2	2	2
Isocitrate lyase	1	1	1	1
Immune modulation	29	38	36	40
Motility	3	3	3	3
Nutritional/Metabolic factors	27	58	58	41
Response regulation	2	2	2	3
Stress survival	4	5	5	5
**Percentage of 'Plant only interaction factors' (PIFAR)**				
Detoxification	9	8	9	8
EPS	13	10	12	13
LPS	3	2	2	2
PCWDE	3	2	2	2
Toxins	38	39	39	37
**Percentage of 'Plant growth-promoting traits (PGPT)**				
Bio-fertilisation	14	14	14	14
Bio-remediation	8	9	9	8
Colonising plant system	31	31	31	32
Competitive exclusion	19	18	19	19
Phytohormone/plant signal production	8	9	8	9
Stress control/biocontrol	18	18	18	18

Supplementary Table 5: Positive and variable utilisation of carbon sources by current members of the genus *Raoultella*.

Characteristic	***Raoultella scottia***	***Raoultella lignicola***	***Raoultella terrigena***	***Raoultella planticola***	***Raoultella electrica***	***Raoultella ornitholytica***
**Utilisation of (Biolog):**						
Dextrin	+	+	+	+	+	+
D-cellobiose	+	+	+	+	+	+
Gentiobiose	+	+	+	+	+	+
D-raffinose	+	+	+	+	+	+
α-D-lactose	+	+	+	+	+	+
D-melibiose	+	+	+	+	+	+
D-salicin	+	+	+	+	+	+
N-acetyl-D-glucosamine	+	+	+	+	+	+
N-acetyl-D-manosamine	+	+	+	+	+	+
α-D-glucose	+	+	+	+	+	+
D-mannose	+	+	+	+	+	+
D-fructose	+	+	+	+	+	+

D-galactose	+	+	+	+	+	+
3-methyl glucose	+	+	+	+	+	+
L-fucose	+	+	+	+	+	+
L-rhamnose	+	+	+	+	+	+
D-mannitol	+	+	+	+	+	+
myo-inositol	+	+	+	+	+	+
D-glucose-6-phosphate	+	+	+	+	+	+
D-fructose-6-phosphate	+	+	+	+	+	+
D-serine	+	+	+	+	+	+
L-arginine	+	+	+	+	+	+
L-histidine	+	+	+	+	+	+
L-serine	+	+	+	+	+	+
Pectin	+	+	+	+	+	+
D-galacturonic acid	+	+	+	+	+	+
L-galactonic acid lactone	+	+	+	+	+	+
D-gluconic acid	+	+	+	+	+	+
D-glucuronic acid	+	+	+	+	+	+
Mucic acid	+	+	+	+	+	+
D-saccharic acid	+	+	+	+	+	+
Citric acid	+	+	+	+	+	+
L-malic acid	+	+	+	+	+	+
**Variable reactions to (Biolog):**						
D-maltose	v				+	+

D-trehalose	+		+	+	+	+
Sucrose	+		+	+	+	+
D-turanose	v	+	+	+	+	
Stachyose	v	+		+	+	+
β-methyl-D-glucoside	+		+	+	+	+
N-acetyl-D-galactosamine	v	+	+	+	+	+
N-acetyl neuraminic acid	v	+	+	+	+	+
D-fucose	+	+	+	+	+	
Inosine	v	+	+	+	+	+
D-sorbitol	+		+	+	+	+
D-arabitol	v	+		+	+	+
Glycerol	v	+		+	+	+
D-aspartic acid		+				
Gelatin	v	+	+		+	
Glycyl-L-proline	v	+	+	+	+	+
L-alanine	v	+	+	+	+	+
L-aspartic acid	v	+	+	+	+	+
L-glutamic acid	v	+	+	+	+	+
L-pyroglutamic acid		+				
Glucuronamide	+	+	+		+	+
Quinic acid	+	+	+	+	+	
p-hydroxy-phenylacetic acid	v	+	+	+	+	+
Methyl pyruvate	v	+		+	+	+

D-lactic acid methyl ester		+		+	+	
L-lactic acid	v	+	+	+	+	+
D-malic acid	v	+	+	+		+
Bromo-succinic acid	v	+		+	+	+
Tween 40		+	+		+	
γ-amino-butyric acid		+		+		+
β-hydroxy-D,L-butyric acid		+	+	+	+	+
Acetoacetic acid		+	+	+	+	
Acetic acid	v	+	+	+	+	+
Formic acid	v	+	+	+	+	+

+, 90 – 100 % strains +; v, variable;

Supplementary Figure 1: Maximum likelihood tree based on the almost complete 16S rRNA gene sequences (1,389 bp) of *Raoultella scottia* sp. nov., *Raoultella lignicola* sp. nov. and the closest phylogenetic relatives. Bootstrap support values higher than 50 % after 1000 replicates are shown next to the branches and *Cronobacter sakazakii* was used as an outgroup. The fraction of substitutions per site is indicated by the scale bar.

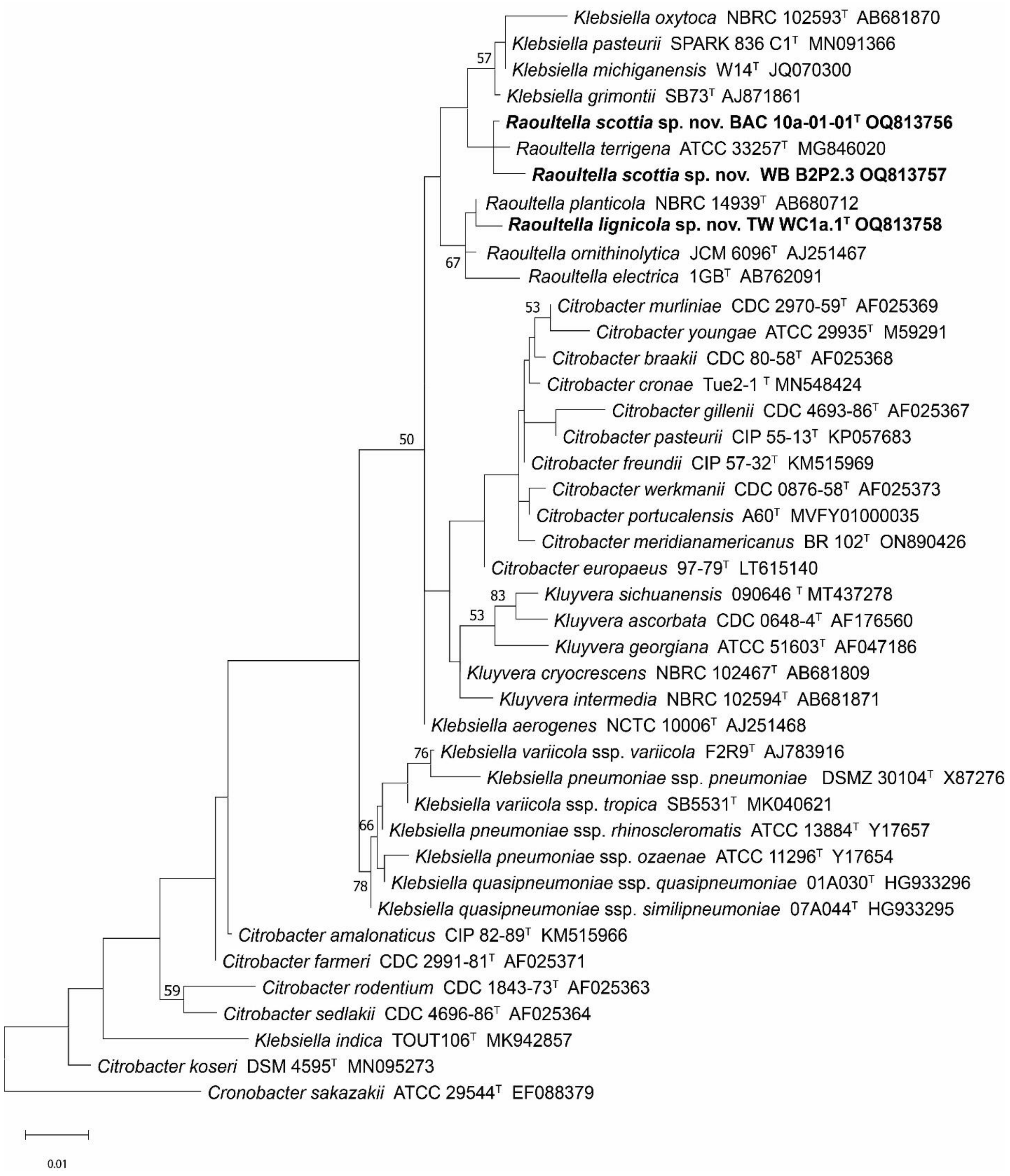
Klebsiella oxytoca NBRC 102593^{T} AB681870
Klebsiella pasteurii SPARK 836 C1^{T} MN091366
57
Klebsiella michiganensis W14^{T} JQ070300
Klebsiella grimontii SB73^{T} AJ871861
Raoultella scottia sp. nov. BAC 10a-01-01^{T} OQ813756
Raoultella terrigena ATCC 33257^{T} MG846020
Raoultella scottia sp. nov. WB B2P2.3 OQ813757
Raoultella planticola NBRC 14939^{T} AB680712
Raoultella lignicola sp. nov. TW WC1a.1^{T} OQ813758
Raoultella ornithinolytica JCM 6096^{T} AJ251467
67
Raoultella electrica 1GB^{T} AB762091
53
Citrobacter murliniae CDC 2970-59^{T} AF025369
Citrobacter youngae ATCC 29935^{T} M59291
Citrobacter braakii CDC 80-58^{T} AF025368
Citrobacter cronae Tue2-1 ^{T} MN548424
Citrobacter gillenii CDC 4693-86^{T} AF025367
50
Citrobacter pasteurii CIP 55-13^{T} KP057683
Citrobacter freundii CIP 57-32^{T} KM515969
Citrobacter werkmanii CDC 0876-58^{T} AF025373
Citrobacter portucalensis A60^{T} MVFY01000035
Citrobacter meridianamericanus BR 102^{T} ON890426
Citrobacter europaeus 97-79^{T} LT615140
83
Kluyvera sichuanensis 090646 ^{T} MT437278
53
Kluyvera ascorbata CDC 0648-4^{T} AF176560
Kluyvera georgiana ATCC 51603^{T} AF047186
Kluyvera cryocrescens NBRC 102467^{T} AB681809
Kluyvera intermedia NBRC 102594^{T} AB681871
Klebsiella aerogenes NCTC 10006^{T} AJ251468
76
Klebsiella variicola ssp. variicola F2R9^{T} AJ783916
Klebsiella pneumoniae ssp. pneumoniae DSMZ 30104^{T} X87276
Klebsiella variicola ssp. tropica SB5531^{T} MK040621
66
Klebsiella pneumoniae ssp. rhinoscleromatis ATCC 13884^{T} Y17657
Klebsiella pneumoniae ssp. ozaenae ATCC 11296^{T} Y17654
78
Klebsiella quasipneumoniae ssp. quasipneumoniae 01A030^{T} HG933296
Klebsiella quasipneumoniae ssp. similipneumoniae 07A044^{T} HG933295
Citrobacter amalonaticus CIP 82-89^{T} KM515966
Citrobacter farmeri CDC 2991-81^{T} AF025371
59
Citrobacter rodentium CDC 1843-73^{T} AF025363
Citrobacter sedlakii CDC 4696-86^{T} AF025364
Klebsiella indica TOUT106^{T} MK942857
Citrobacter koseri DSM 4595^{T} MN095273
Cronobacter sakazakii ATCC 29544^{T} EF088379
0.01

Supplementary Figure 2: Transmission electron microscopy of a) *Raoultella scottia* sp. nov. BAC 10a-01-01^{T} and b) *Raoultella lignicola* sp. nov. TW_WC1a.1^{T} displaying the fimbriae. Scale bar, 1 µm and 500 nm.

a)

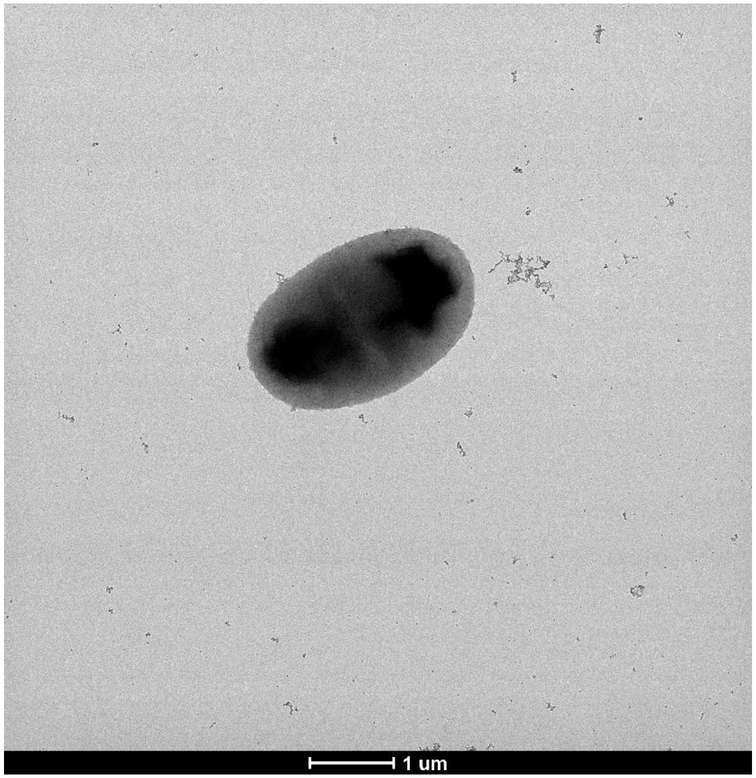

b)

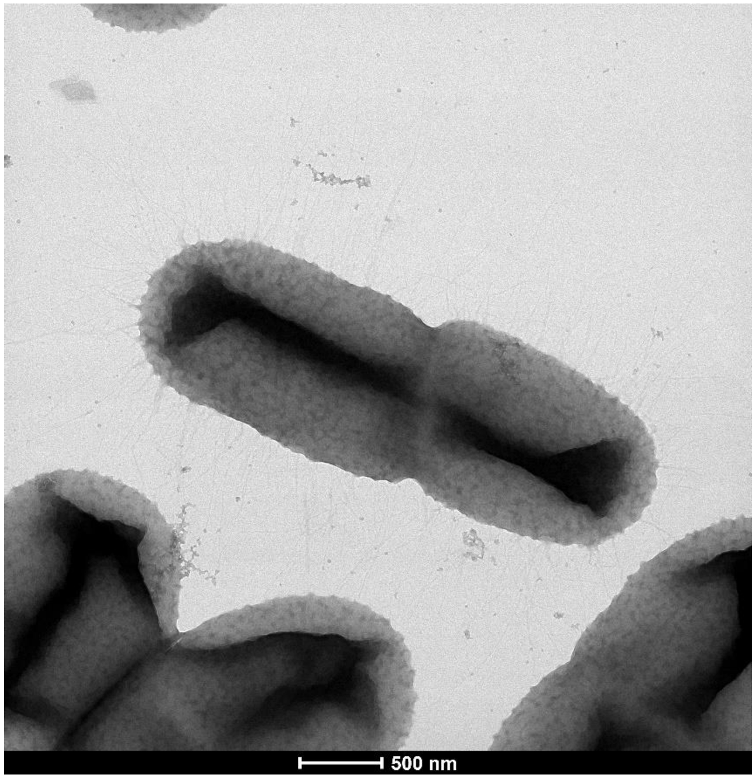

Supplement: Supplementary file 1 [file Data_Sheet_1.pdf]
